# Surgical site infection prevention in Gynae-oncology unit: together we can

**DOI:** 10.1017/ash.2025.138

**Published:** 2025-09-03

**Authors:** Ke Juin Wong, Suguna A/P Subramanian, Maria Limjoon, Cynthi Christie Anthony, Prisca Masiry, Siew Moy Fong

**Affiliations:** 1 Head of Infection control unit, HWKKS, Sabah, Malaysia; 2 Head of Gynae-oncology unit, HWKKS, Sabah, Malaysia; 3 Infection control nurse, Infection control unit, HWKKS, Sabah, Malaysia; 4 Infection control nurse, Infection control unit, HWKKS, Sabah, Malaysia; 5 Clinical nurse, Gynae-oncology unit, HWKKS, Sabah, Malaysia; 6 Infectin control Consultant, HWKKS, Sabah, Malaysia

## Abstract

**Objective:** To describe a collaboration effort between gynae-oncology and infection control unit in a sustainable surgical side infection prevention program **Methods:** In January 2023, gynae-oncologist noted a surge in surgical side infection (SSI) in gynae-oncology unit in Sabah Women and Children’s Hospital (SWACH), Kota Kinabalu, Sabah, Malaysia. The increasing trend of SSI was further confirmed by active surveillance started in January and February 2023. The SSI rate was found to be up to 46.2% (6 out of 13) in the elective gynae-oncology cases and 5 out of 15ases (31.2%) in February 2023.

Outbreak interventions taken place. A combined continuous medical education of the latest SSI guidelines was carried out in the gynae-oncology unit including clinical nurses, clinicians and infection control team (ICT).

Ward clinical nurses and infection control nurses developed SSI prevention program based on the latest SSI guideline and started ward clinical nurse education. An active SSI surveillance team was formed consisting ward sister and one clinical nurse, chief clinicians and infection control nurse to collect SSI cases. **Results:** SSI rate had reduced and maintained since March 2023. The SSI rate was maintained at zero except June and August with one superficial SSI respectively. Since September until December 2023 there was no SSI detected in active surveillance. **Conclusions:** Collaborative effort and understanding between clinical services and infection control unit are important in creating an effective and sustainable infection prevention program. Effective infection prevention program is not necessarily expensive. In fact, a highly motivated team, simple and practical approach can have amazing results.

